# Prediction of Type 2 Diabetes Mellitus From Chest X-Rays Using a Suite of Previously Developed Chronic Disease Deep Learning Models in an Ethnically Diverse Cohort: Observational Study

**DOI:** 10.2196/85248

**Published:** 2026-07-03

**Authors:** Pola Lydia Lagari, Awais Farooq, Brian Thomas Layden, Ayis Pyrros, William Galanter

**Affiliations:** 1 Department of Medicine University of Illinois Chicago Chicago, IL United States; 2 Medical University of South Carolina Charleston, SC, SC United States; 3 Duly Health and Care, Department of Radiology, Downers Grove, IL Chicago, IL United States; 4 Department of Biomedical and Health Information Sciences University of Illinois Chicago Chicago, IL United States

**Keywords:** type 2 diabetes, multimodal machine learning, chest x-rays, deep learning, risk assessment, neural networks, risk prediction

## Abstract

**Background:**

Screening for type 2 diabetes (T2D) is not optimal, leading to a large number of patients being undiagnosed. Recently, deep learning (DL) applied to chest radiographs (CXRs) has shown promise for opportunistic T2D prediction. A prior study in a predominantly suburban non-Hispanic White cohort achieved an area under the curve (AUC) of 0.84 for prevalence. In this study, we evaluate the performance and generalizability of this DL model in an urban cohort with greater racial diversity, higher social deprivation, and higher T2D prevalence. We further assess whether integrating DL predictions with BMI and demographic variables improves T2D prediction beyond demographics and BMI alone.

**Objective:**

This study aims to externally validate a previously developed DL-based CXR model for T2D prediction in a diverse urban population, to assess its performance for both prevalent and incident T2D, and to determine whether combining DL predictions with demographics and BMI improves predictive performance.

**Methods:**

We studied adults (2010-2020) from a tertiary academic medical center in Chicago with at least one ambulatory CXR. First, we performed external validation of a previously developed DL-CXR model by applying it directly to our cohort. Second, we evaluated whether combining the DL model output with additional data, demographics, BMI, and social deprivation index improved the performance. T2D prevalence was modeled using extreme gradient boosting, while incidence was assessed with Cox proportional hazards models. Model performance was compared using AUC and concordance, and feature contributions were evaluated using feature importance and odds ratios.

**Results:**

Among 39,908 patients (n=21,311, 53.4% non-Hispanic Black; n=9179, 23% Latino; and n=5587, 14% non-Hispanic White), 26% (n=10,376) had T2D at their first CXR. The previously developed DL-T2D model maintained discrimination for prevalent T2D in this diverse urban cohort, with similar performance across racial groups (Latino: 0.818; non-Hispanic White: 0.819; non-Hispanic Black: 0.790), supporting generalizability. Adding DL output to demographics and BMI improved prediction compared with clinical variables alone (AUC 0.808 vs 0.766; *P*<.001). For a 3-year incident T2D, the full model achieved an AUC of 0.709 with concordance of 0.707; individuals in the highest risk quartile had a 7-fold higher incidence.

**Conclusions:**

In a diverse urban cohort, a previously developed DL model applied to CXRs provided significant incremental value beyond demographics and BMI for T2D risk prediction. Despite substantial differences in population characteristics compared with the derivation cohort, the DL model remained effective for T2D screening. Incidence prediction was less accurate than prevalence, highlighting the need for further refinement, potentially incorporating hemoglobin A_1c_ when available. Although racial disparities in prevalence exist, predictive performance was comparable across groups. These findings support the generalizability of CXR-based DL for opportunistic T2D screening in diverse populations.

## Introduction

Type 2 diabetes (T2D) is a prevalent chronic disease with significant public health implications, especially in populations facing socioeconomic and health care disparities. According to the Centers for Disease Control and Prevention, approximately 11.6% of the US population has been diagnosed with diabetes, affecting 38.4 million Americans. Effective screening of T2D is key to mitigating complications. Undiagnosed diabetes affects 1-2% of US adults, accounting for roughly 10% of all cases [1]. Prevalence of undiagnosed diabetes increases with age, rising from 1.3% in those aged 20-39 years to 6.8% in those over 60 [2]. The gap in early detection is often exacerbated by limited access to health care, inconsistent screening practices, and inadequate awareness of the risk factors among patients and clinicians [3-5].

Opportunistic screening [6], which leverages data collected during unrelated clinical care, holds promise for addressing this issue. By using existing data, such as imaging or blood tests, clinicians can identify patients at high risk of T2D, thereby increasing the likelihood of early detection, particularly in populations that may not seek diabetes screening.

Recent advancements in artificial intelligence, specifically deep learning (DL) models, offer innovative approaches to disease prediction using imaging data [7,8], such as chest radiographs (CXRs), computed tomography (CT), and retinal images. Integrating DL with CT imaging and clinical data has improved prognostic performance in assessing COVID-19 disease severity [9]. A foundation model, RETFound, has been adapted to outperform comparative models in diagnosing sight-threatening eye diseases [10]. Moreover, DL models analyzing retinal photographs have accurately predicted adverse cardiovascular events, achieving performance comparable to traditional risk assessments [11]. Previous research [12] demonstrated the feasibility of predicting T2D using DL models that combine CXRs with clinical data, achieving an AUC of 0.84 (95% CI 0.83-0.85). However, as health care systems serve populations with varying diversity, assessing whether these models maintain performance across different demographic groups is critical.

## Methods

### Study Design and Population

This study is a retrospective cohort analysis conducted at an urban, tertiary academic medical center in Chicago, UI-Health, including a hospital, on-site and off-site clinics, as well as a Federally Qualified Health Center (FQHC). We included adult patients (≥18 years old) who had at least one upright, ambulatory CXR between January 1, 2010, and September 12, 2020, and at least one prior visit with a billing clinician. Patients were excluded if they had missing demographic information or had type 1 DM.

### Data Collection and Variables

CXR images were retrieved from a research data warehouse maintained by the UI-Health Center for Clinical and Translational Science (CCTS). Age, sex, race, ethnicity, BMI, zip code, hemoglobin A_1c_ (HbA_1c_), and prescribed Diabetes medications (Multimedia Appendix 1) were retrieved from the electronic health record (EHR). The dates and *International Classification of Diseases 9 (ICD-9)* and *International Classification of Diseases 10* (*ICD-10*) diagnostic codes for T2D and type 1 diabetes (Multimedia Appendix 2) were extracted from an archived copy of our prior EHR. The social deprivation index (SDI) [13] was derived from zip codes.

DL CXR risk models were previously developed and published for use with ambulatory CXRs [12]. These models were based on a derivation cohort, Table 1 of CXRs for diagnoses based on Medicare’s risk adjustment model using hierarchical condition categories (HCC) [14]. These included HCC18 (diabetes with chronic complications), HCC22 (morbid obesity), HCC85 (congestive heart failure), HCC96 (specified heart arrhythmias), HCC108 (vascular disease), and HCC111 (chronic obstructive pulmonary disease). These models are noted as DL-T2D, DL-Obesity, DL-CHF (congestive heart failure), DL-Arrhythmias (specified heart arrhythmias), DL-Vasc (vascular disease), and DL-COPD (chronic obstructive pulmonary disease). DL-T2D is the model used in our prior publication, for which our cohort serves as an external test.

**Table 1:** Population statistics for prevalence, incidence, and derivation cohorts.

Feature^a^	Prevalence	Incidence	Derivation^b^
Unique patients, n	39,908	19,671	153,168
Age (years), mean (SD)	46.5 (16.8)	41.3 (16.42)	56 (18)
Female, n (%)	24,005 (60.15)	12,776 (64.95)	84,089 (54.9)
SDI^c^, median (IQR)	79.9 (68-97)	80.5 (68-97)	18 (8-35)
BMI, mean (SD)	30.8 (8.60)	30.18 (8.31)	29.7
Asian, n (%)	1317 (3.3)	688 (3.5)	9650 (6.3)
Non-Hispanic Black, n (%)	21,311 (53.4)	11,253 (57.2)	8577 (5.6)
Latino, n (%)	9179 (23)	3836 (19.5)	10,262 (6.7)
Non-Hispanic White, n (%)	5587 (14)	2754 (14)	114,264 (74.6)
Other, n (%)	2514 (6.30)	1140 (5.8)	10,415 (6.8)
T2D^d^, n (%)	10,376 (26)	3147 (16)	29,255 (19.1)
Follow-up (days), median (IQR)	—^e^	965 (428-1839)	—

^a^Population statistics for prevalence, incidence, and derivation cohorts in a retrospective study of T2D. The study included adults aged ≥18 years who underwent ambulatory chest radiography at UI Health in Chicago, between January 1, 2010, and September 12, 2020. Prevalence and incidence were derived from data of patients from UI Health, and the data from the derivation cohort are from [12].

^b^For the T2D proportion in the incidence cohort, the measure is all the patients who did not have T2D at the time of chest radiograph but developed T2D during follow-up [12].

^c^SDI: social deprivation index.

^d^T2D: type 2 diabetes.

^e^Not applicable.

### Outcome Ascertainment

The primary outcome was the diagnosis of T2D, requiring at least one of the following: *ICD-9* or *ICD-10* diagnosis code for T2D (Multimedia Appendix 2) or HbA_1c_ ≥ 6.5% or a prescription for diabetes-specific medication (Multimedia Appendix 1). In Multimedia Appendix 3, we show the percentage of the cohort identified with T2D based on medications, HbA_1c_ ≥ 6.5%, and *ICD-9* and *ICD-10* codes. To determine the prevalence of T2D, we searched for evidence prior to the CXR, assuming T2D is a chronic disease. For the incidence cohort, the diagnosis needed to occur after the CXR.

### Model Development and Analysis

To predict T2D prevalence and incidence, we developed and evaluated several models combining DL methods with traditional clinical and demographic variables.

### Prevalence Prediction

We used extreme gradient boosting (XGBoost) models to predict T2D prevalence. We randomly split the dataset into training and test sets using an 80-20 ratio, applying a stratified sampling method to ensure that the class distribution remained consistent across both subsets. All models were trained on the same 80% training set and evaluated on the same 20% held-out test set to ensure comparability. We built 6 different models.

Model 1 (demographics: age, race, SDI, and sex), model 2 (BMI), model 3 (BMI and demographics), model 4 (DL-T2D, the derivation model being tested), model 5 (BMI, demographics, and DL-T2D), model 6 (all variables). All models were trained and evaluated on the same data split for comparability.

**Figure 1:**
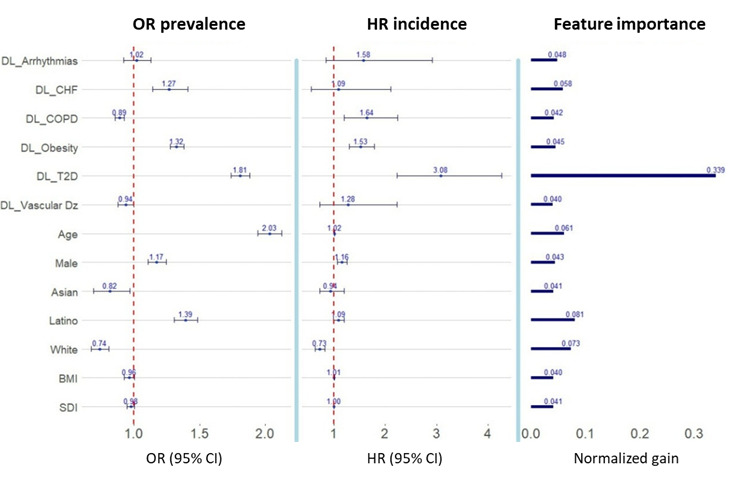
Forest plot of extreme gradient boosting feature importance and logistic regression ORs for prevalence and Cox proportional HRs for incidence. Multivariable model results for predicting T2D prevalence and incidence in a retrospective cohort study of adults aged ≥18 years who underwent ambulatory chest radiography at a tertiary academic medical center (UI Health) in Chicago, Illinois, between January 1, 2010, and September 12, 2020. The first panel shows a forest plot of ORs and 95% CIs from the fully adjusted logistic regression model for T2D prevalence. ORs were normalized such that a 1-unit change corresponds to an SD of each continuous variable. The middle panel shows HRs and 95% CIs from the fully adjusted Cox proportional hazards regression model for T2D incidence. The last panel displays feature importance for the XGBoost model predicting T2D prevalence. Female sex and non-Hispanic Black race were used as reference categories as the most common values in their respective categories and are, therefore, not shown in the figure. All models include demographic variables, BMI, and DL-derived imaging biomarkers unless otherwise specified. CHF: congestive heart failure; COPD: chronic obstructive pulmonary disease; DL: deep learning; HR: hazard ratios; OR: odds ratio; SDI: social deprivation index; T2D: type 2 diabetes.

We assessed model performance using the area under the curve (AUC) for the receiver operating characteristic (ROC). We compared the AUC of all these models in Table 2. We also show the feature importance and odds ratios (ORs) values of model 4 for the prevalence cohort in Figure 1.

Hyperparameter selection for the XGBoost models was performed using a structured search strategy on the training set. For each hyperparameter combination, model performance was assessed using 5-fold cross-validation within the training set, with 15% of the training set held out as a validation fold in each iteration. We evaluated combinations of key hyperparameters, including maximum tree depth (range 2-4), learning rate (0.01-0.1), L1 (alpha: 0-0.5), and L2 (lambda: 0.5-2) regularization strengths. Model performance was assessed using AUC on the validation set, with emphasis on selecting configurations that balanced predictive performance and model simplicity to mitigate overfitting. We constrained model complexity through shallow trees and regularization. The objective function is binary logistic loss. To address class imbalance, we applied *scale_pos_weight* proportional to the ratio of negative to positive samples in the training set.

The final model parameters (maximum depth=4, learning rate=0.01, alpha=0.1, lambda=1) were chosen as they provided the best mean AUC in cross-validation.

Regarding missing data, patients with missing demographic information were excluded. No additional missing data were present in the remaining covariates used for modeling.

For probability calibration, we applied decile-based calibration and assessed agreement between predicted and observed risk across deciles.

The classification threshold was determined using Youden’s J statistic derived from the ROC curve calculated on the training set only, and this fixed threshold was subsequently applied to the test set for performance evaluation.

In order to make the OR more meaningful and consistent, for each continuous variable, the data were normalized to set the mean to 0 and the SD to 1. Therefore, in Table 2 and Figure 1, the ORs represent a 1 SD increase in the value of the covariate.

**Table 2:** Area under the curve (AUC) and concordance for prevalence and incidence test cohorts.

Models^a^	Prevalence	Incidence			
	AUC^b^ (95% CI)	AUC (3 years)^c^, (95% CI)	AUC (5 years) ^c^, (95% CI)	AUC (7 years) ^c^, (95% CI)	Concordance^d^, (95% CI)
External validation of prior DL^e^ model [12]^f^	0.792 (0.781-0.802)	0.695 (0.682-0.707)	0.683 (0.670-0.696)	0.666 (0.650-0.681)	0.680 (0.676-0.685)
DL_Obesity	0.652 (0.646-0.658)	0.569 (0.537-0.601)	0.589 (0.558-0.620)	0.588 (0.552-0.623)	0.585 (0.579-0.591)
Demographics (Dem)	0.744 (0.732-0.755)	0.664 (0.644-0.685)	0.643 (0.621-0.665)	0.628 (0.601 -0.654)	0.656 (0.640-0.664)
BMI	0.609 (0.596-0.623)	0.560 (0.528-0.572)	0.562 (0.539-0.586)	0.570 (0.543-0.597)	0.564 (0.547-0.581)
Dem+BMI	0.766 (0.755-0.777)	0.677 (0.656-0.697)	0.666 (0.644-0.687)	0.647 (0.621-0.673)	0.674 (0.658-0.689)
Dem + DL-T2D^g^ + BMI	0.808 (0.798-0.818)	0.703 (0.684-0.723)	0.692 (0.671-0.714)	0.672 (0.647-0.698)	0.697 (0.682-0.712)
Dem + all DL + BMI	0.817 (0.807-0.827)	0.709 (0.689-0.720)	0.699 (0.679-0.720)	0.681 (0.655-0.706)	0.707 (0.692-0.722)

^a^Performance of clinical and DL models for prediction of T2D prevalence and incidence in a retrospective study of adults aged ≥18 years who underwent ambulatory chest radiography at UI Health in Chicago, between January 1, 2010, and September 12, 2020. Model performance is supported using AUC and concordance metrics. Models evaluated include: the previously published DL-T2D [12]; demographics-only variables; BMI alone; demographics plus BMI; the DL-based T2D model (DL-T2D) alone; a combined model including demographics, BMI, and DL-T2D; and a comprehensive model incorporating demographics, BMI, and multiple DL-derived imaging biomarkers (T2D, morbid obesity, congestive heart failure, arrhythmia, chronic obstructive pulmonary disease, and vascular disease). This comparison illustrates the incremental contribution of clinical variables and DL-derived features to T2D prediction performance.

^b^AUC determined from extreme gradient boosting (XGBoost).

^c^AUC from Cox proportional hazard model and timeROC.

^d^Concordance from Cox proportional hazard model.

^e^DL: deep learning.

^f^Test of prior model on 100% of prevalence and incidence cohorts; the other models tested on a 20% sample.

^g^T2D: type 2 diabetes.

### Incidence Analysis

#### Kaplan-Meier Survival Analysis

**Figure 2:**
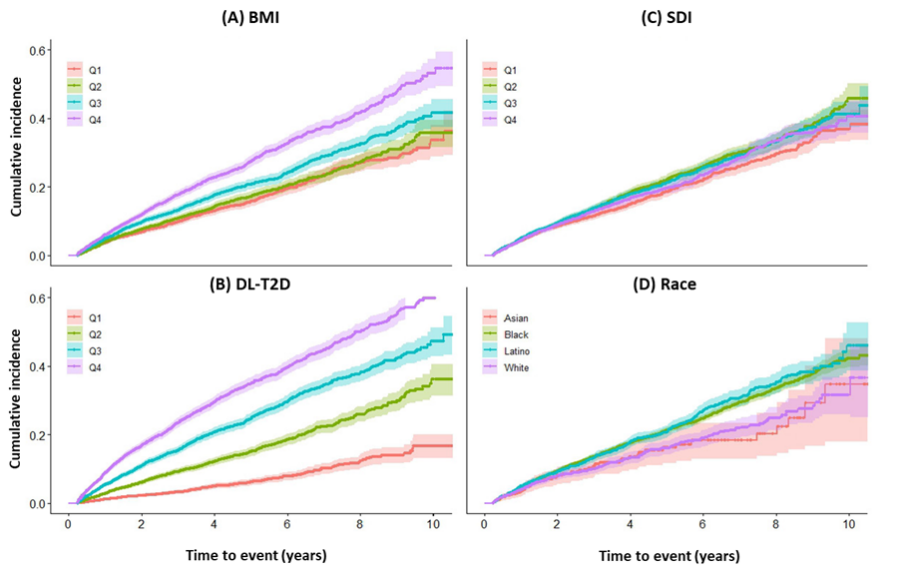
Kaplan-Meier curves of the incidence of T2D by quartile of covariate. It is the cumulative incidence of T2D estimated using Kaplan-Meier survival analysis in a retrospective cohort study of adults aged ≥ 18 years undergoing ambulatory chest radiography at a tertiary academic medical center in Chicago, Illinois, between January 1, 2010, and September 12, 2020. The incidence cohort included individuals without prevalent T2D at the time of the index chest radiograph and with available follow-up encounters; patients diagnosed with T2D or lost to follow-up within the first 90 days were excluded. Cumulative T2D incidence is shown stratified by quartiles of (A) BMI; (B) DL-T2D, a risk score derived from a DL model applied to chest radiographs; (C) SDI; and (D) self-reported race. Follow-up continued until T2D diagnosis, last clinical encounter, or end of the study period. The figure demonstrates differences in incidence across the four variables, each split into 4 quartiles. DL-T2D shows the greatest dispersion: at 5 years, the risk for Q4 is about 40%, while for Q1, it is <10%, with risk increasing progressively across quartiles. BMI also shows some dispersion of risk, but less than DL-T2D. The SDI shows minimal dispersion and some overlap of the curves of the different quartiles. This cohort shows little variability, with results near the maximum value, limiting its usefulness for examining the role of the SDI. Risk stratification by race shows a higher incidence among Latino and non-Hispanic Black patients, whereas non-Hispanic White and Asian patients have a lower risk. DL: deep learning; SDI: social deprivation index; T2D: type 2 diabetes.

Kaplan-Meier survival curves were generated for the incidence cohort to estimate the probability of T2D incidence over time, stratified by covariate quartiles, and are shown in Figure 2 [15,16].

#### Cox Proportional Hazards Models

We used Cox proportional hazards (CPH) models to evaluate the relationship between variables and time to T2D diagnosis. Patients were censored at the date of their last visit to ensure only active observation data inclusion. This approach accounted for patients lost to follow-up. Metrics derived from the CPH models included concordance [17], variable hazard ratios (HRs), and time-dependent AUC at specific time points using the *timeROC* package in R [18].

HRs were determined from the coefficients of the CPH models. HRs were normalized in the same manner as ORs described above.

#### Incidence AUC Evaluation Versus Time

AUC was assessed at 3, 5, and 7 years to examine the model’s ability to predict incident T2D over time using *timeROC* [18], which uses inverse propensity weighting [19] to minimize censoring bias.

#### Association of Covariates to Calibration and AUC

We produced calibration plots to investigate any changes in calibration by race, age, or BMI in Multimedia Appendix 4. Race was examined by considering the following: Asian, non-Hispanic Black, Latino, and non-Hispanic White. For age and BMI, calibration plots were generated for the ranges of each variable. For ages: 18-45, 45-65, and >65 years. For BMI, we looked at normal (<25), overweight (25-30), and obese (>30).

For the prevalence, the AUC’s CI was determined using DeLong variance estimate [20]. For the incidence AUCs, the CIs were performed with *timeROC*. The concordance values were compared using *CompareC* in the *survcomp* [21] package in R. Statistical significance was set as *P*<.05, and the Holm-Bonferroni method was used to account for multiple testing.

### Ethical Considerations

This study was approved by the Institutional Review Board at the University of Illinois Chicago, with protocol number 2024-0187, on February 26, 2024. The use of de-identified patient data from UI Health for research purposes complied with all regulatory requirements, including the Health Insurance Portability and Accountability Act (HIPAA), ensuring patient privacy and data security. Data included from the prior study [12] were aggregated without any identification.

## Results

### Study Population

**Figure 3:**
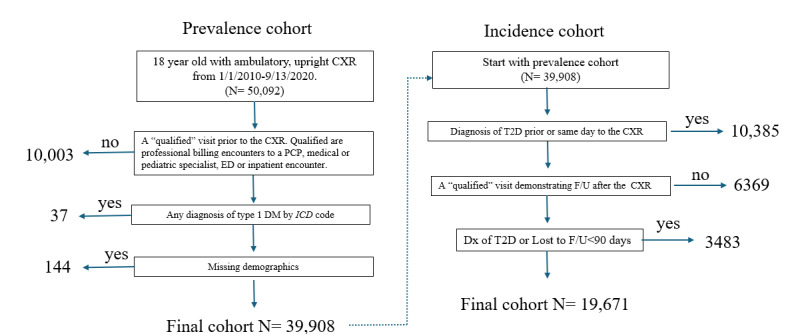
Description of cohorts. Adult patients (≥18 years) with ambulatory CXRs between January 1, 2010, and September 12, 2020, were identified (N=50,092). CXRs were included if associated with qualified encounters (primary care, medical or pediatric specialty, ED, or inpatient visits), and patients with type 1 diabetes were excluded, along with patients with missing demographic data, yielding the final prevalence cohort of 39,908 unique patients. The incidence cohort was derived by excluding patients with T2D diagnosed prior to the index CXRs, as well as all patients who developed T2D or lost follow-up within 90 days after the CXR, to allow for “fresh” hemoglobin A1c and requiring at least one qualified follow-up encounter after CXR, resulting in 19,671 patients. Follow-up continued until T2D diagnosis, last billing encounter, or study end (September 12, 2020). CXR: chest radiograph; DM: diabetes mellitus; Dx: diagnosis; ED: emergency department; F/U: follow-up; ICD: International Classification of Diseases; PCP: primary care provider or physician; T2D: type 2 diabetes.

The characteristics of the cohorts are shown in Table 1. The incidence cohort is slightly younger and has more females than the prevalence cohort, with roughly similar racial and SDI means, and a median follow-up time of about 2 years. The SDI in both study cohorts was significantly higher than that of the derivation cohort. The racial composition of the study cohorts differed substantially from that of the derivation cohort (Figure 3).

### Model Performance

#### Prevalence

We first performed external validation of the previously developed DL-T2D model by applying it directly to our cohort. The DL-T2D model achieved an AUC of 0.792 (95% CI 0.781-0.802) for T2D prevalence.

We then evaluated whether combining DL predictions with clinical variables improved performance. Among models without DL predictors, AUC ranged from 0.609 (95% CI 0.596-0.623) for BMI alone to 0.766 (95% CI 0.755-0.777) for demographics plus BMI. Adding the DL-T2D predictor to demographics and BMI significantly improved discrimination (AUC 0.808, 95% CI 0.798-0.818 vs 0.766, 95% CI 0.755-0.777; *P*<.001), demonstrating incremental value beyond clinical variables alone.

And finally, the addition of multiple DL predictors showed the best performance with an AUC of 0.817 (95% CI 0.807-0.827; *P*<.001). All results are summarized in Table 2.

#### Incidence

For incident T2D prediction, we similarly began by evaluating the externally validated DL-T2D model, which achieved a concordance of 0.680 (95% CI 0.676-0.685). Among clinical models without DL, performance improved stepwise from BMI alone to demographics alone to demographics plus BMI (*P*<.001 for comparisons).

Adding demographics and BMI to DL-T2D resulted in modest additional improvement (0.697, 0.682-0.712). The full model, incorporating all DL predictors along with demographics and BMI, performed better than DL-T2D alone, 0.707 (95% CI 0.692-0.722; *P*=.002). Time-dependent AUCs at 3, 5, and 7 years showed similar comparative patterns, with detailed curves provided in Multimedia Appendix 5.

Figure 1 summarizes the associations of all predictors with disease prevalence and incidence. In the logistic regression model of prevalence in the first panel, age had the strongest association with disease prevalence, followed by DL-T2D, while male sex, Latino race, DL-CHF, and DL-Obesity were also positively associated. The Cox model in the middle panel confirms DL-T2D as the key predictor of disease incidence, with DL-COPD and DL-Obesity also positively associated. Race or ethnicity shows some significance in both models, whereas BMI and SDI had no statistically significant associations. In the XGBoost model in the last panel, DL-T2D dominates feature importance, indicating it contributes the most predictive value among all variables.

Calibration curves and calibration statistics are shown for race, age, and BMI in Multimedia Appendix 4. Age is split into <45, 45-65, and >65 years. BMI is split into <25, 25-30, and > 30. The slope for non-Hispanic White patients is not different than 1, while Asian patients have a slope of 1.26 (1.10-1.44), and Latino patients have a slope of 1.08 (95% CI 1.03-1.13), which are underpredicting. In non-Hispanic Black patients, the slope was 0.94 (95% CI 0.90-0.97).

For age, only >65 years have a slope different than 1, 0.90 (95% CI 0.83-0.97). For BMI <25, 1.10 (95% CI 1.05-1.16), and 25-30, 1.09 (95% CI 1.04-1.14). For >30, the slope is 0.92 (95% CI 0.89-0.95).

The AUC for Asian patients is 0.87 (95% CI 0.85-0.89), for non-Hispanic Black patients 0.79 (95% CI 0.78-0.79), for Latino patients 0.82 (95% CI 0.81-0.83), and for non-Hispanic White patients 0.83 (95% CI 0.81-0.84). Patients in the “Other” category were excluded from these specific racial subgroup analyses. For BMI, <25 has an AUC of 0.83 (95% CI 0.82-0.84), 25-30 has an AUC of 0.81 (95% CI 0.80-0.82), and >30 has an AUC of 0.78 (95% CI 0.77-0.79). For age <45 years, the AUC was 0.80 (95% CI 0.78-0.81), for 45-65 years, 0.74 (95% CI 0.73-0.75), and for >65 years, 0.82 (95% CI 0.76-0.88).

## Discussion

### Principal Findings

We demonstrated that incorporating previously established [12] DL predictors into a model including CXRs, demographics, and BMI significantly improves the prediction of T2D prevalence, with the AUC increasing from 0.766 (95% CI 0.755-0.777) to 0.808 (95% CI 0.798-0.818; *P*<.001), and further to 0.817 (95% CI 0.807-0.827) with all DL predictors. The prior DL-T2D model performed well alone for prevalence, with an AUC of 0.792 (95% CI 0.781-0.802) in our cohort. BMI alone performed substantially worse with an AUC of 0.609 (95% CI 0.596-0.623), supporting that DL-T2D is not simply capturing obesity as a surrogate for T2D. Applying the prior model to our cohort resulted in a decrease in performance, from AUC 0.84 to AUC 0.792. The performance in our cohort was better than in the external test in the original study, AUC 0.77 [12]. This modest reduction in performance between the internal test of the derivation and our cohort is not surprising due to significant differences in cohort characteristics (racial distribution, SDI, and T2D prevalence). These differences highlight a meaningful but limited degradation in performance under real-world external deployment conditions.

For the prediction of incidence, the DL predictors did not perform as well in our cohort with AUCs of 0.695 (95% CI 0.682-0.707) at 3 years as in the derivation, 0.79 (95% CI 0.78-0.79) [12]. Our models are more adept at classifying existing cases of T2D than at predicting its future. These models should be compared to present models of incidence, such as the American Diabetes Association Risk Test [22] or the Framingham Offspring T2D risk score [23], in a cohort with additional elements available: Family history, physical activity, and lab tests including A_1c_, and/or fasting glucose. These elements should be recorded in all patients so the variables are unbiased by who they have been ordered by. Because A_1c_, family history, glucose, and exercise history are not always available, a CXR-based prediction may be valuable for a patient with a CXR lacking all other data. This is typical for cohorts taken from actual routine care.

The anatomic areas used by the DL-T2D model to discern risk for T2D have been discussed previously [12], with a video available. In summary, a “change in central mediastinal adiposity is a primary driver. High predictive values include changes in upper abdominal fat and supraclavicular and rib attenuation” [12].

While BMI was somewhat predictive of the prevalence alone, 0.609 (95% CI 0.596-0.623), the DL-T2D alone was significantly superior, 0.792 (95% CI 0.781-0.802). That was also true in the incidence cohort, 3-year AUC, 0.695 (95% CI 0.682-0.707) compared to 0.560 (95% CI 0.528-0.572). These comparisons show that DL-T2D predictions rely on far more than a patient’s BMI alone to assess T2D risk. Supporting this conclusion is the finding that DL-Obesity is a much weaker predictor of T2D risk (Figure 1).

The analysis of the univariate relationship between covariates and the Kaplan-Meier curves in Figure 2 is illustrative. In Figure 2A, the differences between Q1 and Q2 are minimal, but become much more pronounced between Q3 and Q4, suggesting that the BMI-associated risk increase becomes significant from Q2 to Q3, and even more so from Q3 to Q4. The DL-T2D (Figure 2B) shows a large dispersion of risk for each quartile, demonstrating the potential for a good risk classifier. The SDI (Figure 2C) shows minimal dispersion and some overlap of the curves of the different quartiles. Figure 2D indicates that Latino and non-Hispanic Black populations have relatively higher T2D incidence rates compared to non-Hispanic White and Asian populations, though the dispersion is smaller than seen with DL-T2D.

In the derivation study [12], the 3-year incidence AUC was 0.05 lower than the prevalence, 0.84 down to 0.79. In our present analysis, the incidence 3-year AUC was 0.11 lower than the prevalence, 0.82 down to 0.71. It is interesting that the prevalence is nearly as good as the derivation, but the incidence is significantly lower. One possibility is that the power was insufficient to accurately measure the incidence. This is unlikely, as the incidence cohort comprised 19,671 patients with a median follow-up of 965 days, of whom 16% had a diagnosis of T2D. While the precision is lower after 7-8 years (Multimedia Appendix 5), at 3 years it is stable and acceptable, with a CI of 0.689-0.728.

There are multiple reasons why the incidence might not perform as well as the prevalence. Prevalence reflects cross-sectional imaging features of existing disease, whereas incidence requires predicting future disease in initially disease-free patients, introducing additional uncertainty from follow-up variability, competing risks, and changes over time. Because mortality data were unavailable, death could not be modeled as a competing risk and was treated as non-informative censoring, which may modestly overestimate cumulative incidence and HRs for T2D. These differences are further reflected in the composition of the incidence cohort. Looking at Table 1, we see that the incidence cohort is on average 15 years younger than the derivation cohort. There are also about 10 absolute percent more women in the incidence cohort. These changes could explain at least some of the loss of performance.

Incidence may be a combination of newly diagnosed T2D in those who were underdiagnosed, gradual conversion from pre-DM that was present at the time of the CXR, now becoming T2D, as well as new cases that developed and were diagnosed after the CXR. We cannot determine the proportion of each of these in our cohort, due to a small and biased portion of the cohort that had A_1c_ testing. The amount of A_1c_ testing, the SDI [24], and race [25,26] may contribute to underdiagnosis. Since our cohort differed significantly from the derivation cohort by race and SDI, changes in the prevalence of these different causes of a new T2D diagnosis may occur and contribute to the incidence performance loss.

The last thing to consider is the possibility that the radiographic correlates of having T2D might differ at least partially from those of having T2D risk in some patients. This would allow for a model that is trained on prevalence, not performing as well on incidence. Additionally, many of the patients were followed for more than 2 years, with 25% greater than 4.5 years. It is not clear how long the information on the CXR remains accurate over time.

Retraining or fine-tuning the model specifically for longitudinal prediction could improve its performance on incidence. For example, defining a fixed prediction horizon (eg, 3-5 years) and reframing incidence as a binary outcome would allow supervised training tailored to temporal risk. However, this approach introduces additional challenges, including reduced effective sample size due to loss to follow-up and delayed event accrual, which may limit statistical power and model stability. We hope to learn more about and improve this incidence tool in the future.

Feature and covariate associations with prevalence using feature importance and ORs, along with HRs for incidence (Figure 1), demonstrated the important role of the DL-T2D in predictions. Age was a significant risk factor in logistic regression of prevalence, but not for incidence, and it was also not a strong feature in XGBoost [27]. Compared to non-Hispanic Black patients, non-Hispanic White patients were less likely to have T2D, consistent with epidemiological data. However, Latino patients were more likely to have T2D in our cohort, while the prevalence is reported to be lower in Latino than non-Hispanic Black populations [28].

DL-CHF and DL-Obesity had positive relationships with the predictions, while DL-arrhythmias, DL-COPD, and DL-vascular were either non-associated or slightly negatively associated with the prediction (Figure 1). Many of these diseases are chronic and associated with overlapping etiologies and sequelae, so a role in predicting DL-Obesity and DL-CHF is not surprising.

Recently, the SDI was included in the American Heart Association cardiovascular disease risk calculator [29]. The SDI has been shown to be associated with the risk for T2D in a large population in Germany [30] and in the US Gulf States [31]. It has also been shown to be related to the risk for hypertension [32] and complications of T2D [33,34]. The lack of association with prevalence in multivariate regression and the minimal importance in the XGBoost model may reflect the high mean SDI in our cohort (80) and its relatively narrow IQR (68-97), which limits variability and reduces its usefulness as a predictor in this population. Our patient population has a high mean SDI of 80, notably higher than that of the derivation cohort, 18, and a relatively narrow IQR (68-97), limiting variability within the sample. It is possible that greater variance in SDI values would be necessary to show a significant association. Additionally, SDI was measured at the zip code level and may lack sufficient granularity to capture meaningful heterogeneity in social conditions. While SDI reflects structural neighborhood-level disadvantage, it does not account for individual-level socioeconomic factors or other contextual determinants of T2D risk. Therefore, the absence of a significant association in our analysis should not be interpreted as evidence that social deprivation is unrelated to T2D risk, but rather that zip code–level SDI may be insufficiently granular and underpowered to detect such effects in this setting.

To examine the importance of demographics in accuracy, we produced calibration curves and AUCs for different values in Multimedia Appendix 4.

For race, the calibration slope for non-Hispanic White patients was not different than 1, while Asian and Latino patients had slopes greater than 1, or underprediction of higher risk patients. The non-Hispanic Black patients demonstrated a slope less than 1. It might be expected that White patients have excellent calibration, as the derivation cohort had mostly non-Hispanic White patients. The AUCs varied between Black patients (0.79), Latino patients (0.82), non-Hispanic White patients (0.83), and Asian patients (0.87). It is interesting that while Latino and non-Hispanic Black patients were both overrepresented in our cohort compared to the derivation, the AUC of Latino patients was not different than that of White patients (0.83; *P*=.50), while that of non-Hispanic Black patients was lower than that of non-Hispanic White patients and Latino patients (*P*<.001). The AUC was surprisingly better in Asian patients than in White (*P=*.002) or Latino patients (*P*<.001). Other than the fact that the derivation cohort was predominantly non-Hispanic White, it is not clear why non-Hispanic Black patients had a lower AUC and Asian patients had a higher A_1c_. The diagnosis of T2D requires the development of the disease, the clinician’s index of suspicion, and testing behaviors. All these may be associated with socioeconomics, and our SDI was not able to control for socioeconomic factors. Thus, we cannot exclude socioeconomic factors as the basis for the disparity in racial risk model performance.

For BMI, the calibration slope for normal and overweight patients was both greater than 1, while for the obese patients, the slope was 0.92, suggesting underprediction at higher risk. The AUC for normal BMI was 0.83, for overweight patients 0.81, and for obese patients 0.78. This mild deterioration with increasing BMI is not that large, and an AUC of 0.78 can still produce useful risk assessment. It may be related to the effects of certain locations of adiposity in the CXR that are different in the obese than in the non-obese. Further analysis will need to be done to investigate this issue.

### Limitations

Limitations include the use of billing codes and medication orders to define diabetes, which may occasionally lack accuracy. One probable mechanism for errors with medication data is incomplete medication reconciliation for patients treated at outside institutions. We attempted to minimize this by requiring visits where diabetic medication reconciliation should occur.

It is well described that underdiagnosis of T2D is common and associated with ethnic and socioeconomic variables [24-26]. If we were trying to measure the prevalence and incidence of T2D at the population level, underdiagnosis might be accounted for, but we are looking at individual-level CXRs and cannot assign a patient to have a higher risk based on an assumed probability by race and SDI. This problem may produce calibration by race and SDI that could be off due to differing underdiagnosis rates, though our data suggest only small changes by ethnicity and SDI. In our pragmatic cohort, nonrandom variable A_1c_ testing does not allow for correcting for underdiagnosis.

While the population has a broad mix of ethnicities, socioeconomic status is less diverse, with fewer patients having low SDI. Multimedia Appendix 6 shows a histogram with the distribution of SDI values in the prevalence cohort. Further validation of the CXR-based model in more economically diverse cohorts could strengthen its use and ensure equitable application.

Because this study was restricted to patients with an ambulatory CXR, the resulting cohort may not be fully representative of the broader population at risk for T2D, introducing potential bias. As a retrospective study using EHR data, inclusion in the incidence cohort required at least one qualified follow-up encounter after the CXR to ensure outcome ascertainment. This may underrepresent patients who experienced rapid deterioration, died, or transferred care immediately after imaging, potentially biasing the cohort toward healthier survivors. We were unable to control for these biases, as external follow-up and mortality data were unavailable. These issues may affect the derived metrics, and while they could lead to a slight overestimate of T2D incidence, the impact on the model’s predictive performance is unclear. Based on the demographics of our patient population and the rate of censoring, we estimate that death is not likely to represent more than 5% of the patients who leave the study and would not likely have that large of an effect on the prediction metrics.

### Future Directions

Future work could focus on building a more comprehensive AI model trained on larger, more diverse populations to improve generalizability. Additionally, a direct comparison between CXR-based risk assessments and A1c could help evaluate their relative use for early detection and prognosis. Leveraging CXR screening alongside EHR data offers an opportunity to identify high-risk patients who may benefit from targeted screening outreach, thereby enhancing the efficiency and impact of health care resources in addressing undiagnosed or high-risk cases of T2D.

Beyond T2D, our approach could be extended to other diseases where early detection is crucial for preventing adverse outcomes, such as hypertension, chronic kidney disease, systemic atherosclerosis, and cerebral vascular disease. Expanding our model to these broader applications could enhance preventive care and optimize resource allocation across multiple domains of chronic disease management.

### Conclusions

The previously developed DL model [12] demonstrates strong generalizability, performing nearly as well at predicting T2D diagnosis in a more demographically diverse population as in the derivation cohort (AUC 0.792, 95% CI 0.781-0.802, vs 0.84 in the derivation cohort). We next explored whether incorporating demographic variables and BMI could improve predictive performance. Adding both demographics and BMI to the DL model increased the AUC to 0.808, compared with using demographics alone (AUC 0.744, 95% CI 0.732-0.755), BMI alone (AUC 0.609, 95% CI 0.596-0.623), or a combination of demographics and BMI (AUC 0.766, 95% CI 0.755-0.777). Finally, the inclusion of multiple DL-derived predictors yielded the best performance, achieving an AUC of 0.817 (95% CI 0.807-0.827).

Notably, while BMI is a well-established risk factor for T2D, it alone provides only modest predictive power, and the DL-T2D score and prior model consistently emerge as the most influential predictors, outperforming individual patient characteristics such as age, sex, race, SDI, and BMI. While the model remains effective for classifying existing cases (prevalence), its ability to predict future T2D onset (incidence) is considerably weaker.

These findings reinforce the potential use of the model as a valuable tool for opportunistic T2D screening, while highlighting the need for further refinement to enhance its predictive accuracy for incident T2D.

## Data Availability

The data used in this study originate from UI Health’s electronic health record and imaging systems. The imaging data contain protected health information (PHI) and were not scrubbed, as patient identifiers were required to merge data across clinical systems; therefore, the data cannot be publicly shared. We could potentially share aggregate data by request in some circumstances.

## Appendix

Multimedia Appendix 1 List of medications extracted as indicative of type 2 diabetes from January 1, 2010, to September 12, 2020.

Multimedia Appendix 2 International Classification of Diseases for diabetes mellitus.

Multimedia Appendix 3 Euler diagram showing the percentage of the cohort identified with type 2 diabetes based on medications (Multimedia Appendix 1), hemoglobin A1c≥6.5%, and International Classification of Diseases codes (Multimedia Appendix 2). Overlapping regions indicate patients meeting multiple criteria.

Multimedia Appendix 4 Calibration curves (A) and measures (B) for prevalence by age, BMI, and race.

Multimedia Appendix 5 Area under the curve for all variable model (20% test) vs years after chest radiographs.

Multimedia Appendix 6 Distribution of the social deprivation index in the prevalence cohort is shown.

## Abbreviations

AI: artificial intelligence
AUC: area under the curve
CHF: congestive heart failure
COPD: chronic obstructive pulmonary disease
CPH: Cox proportional hazard
CXR: chest radiograph
DL: deep learning
ECE: estimated calibration error
EHR: electronic health record
HbA1c: hemoglobin A1c
HCC: hierarchical condition categories
HIPAA: Health Insurance Portability and Accountability Act
HR: hazard ratio
*ICD-9*: International Classification of Diseases 9
*ICD-10*: International Classification of Diseases 10
OR: odds ratio
SDI: social deprivation index
T2D: type 2 diabetes
XGBoost: extreme gradient boosting

## References

[1] Fang M, Wang D, Coresh J, Selvin E (2022). Undiagnosed diabetes in U.S. adults: prevalence and trends. Diabetes Care.

[2] Gwira JA, Fryar CD, Gu Q (2024). Prevalence of total, diagnosed, and undiagnosed diabetes in adults: United States, August 2021-August 2023. NCHS Data Brief.

[3] American Diabetes Association (2004). Screening for type 2 diabetes. Diabetes Care.

[4] Heianza Y, Hara S, Arase Y, Saito K, Fujiwara K, Tsuji H, Kodama S (2011). HbA1c 5·7-6·4% and impaired fasting plasma glucose for diagnosis of prediabetes and risk of progression to diabetes in Japan (TOPICS 3): a longitudinal cohort study. Lancet.

[5] Zhang Xuanping, Gregg EW, Williamson DF, Barker LE, Thomas W, Bullard KM, Imperatore G (2010). A1C level and future risk of diabetes: a systematic review. Diabetes Care.

[6] Pickhardt PJ, Summers RM, Garrett JW, Krishnaraj A, Agarwal S, Dreyer KJ, Nicola GN (2023). Opportunistic screening: scientific expert panel. Radiology.

[7] Tiu E, Talius E, Patel P, Langlotz CP, Ng AY, Rajpurkar P (2022). Expert-level detection of pathologies from unannotated chest X-ray images via self-supervised learning. Nat Biomed Eng.

[8] Rajpurkar P, Irvin J, Zhu K, Yang B, Mehta H, Duan T, Ding D (2017). CheXNet: radiologist-level pneumonia detection on chest X-rays with deep learning. arXiv.

[9] Lassau N, Ammari S, Chouzenoux E, Gortais H, Herent P, Devilder M, Soliman S (2021). Integrating deep learning CT-scan model, biological and clinical variables to predict severity of COVID-19 patients. Nat Commun.

[10] Zhou Y, Chia MA, Wagner SK, Ayhan MS, Williamson DJ, Struyven RR, Liu T (2023). A foundation model for generalizable disease detection from retinal images. Nature.

[11] Syed MG, Trucco E, Mookiah MRK, Lang CC, McCrimmon RJ, Palmer CNA, Pearson ER (2025). Deep-learning prediction of cardiovascular outcomes from routine retinal images in individuals with type 2 diabetes. Cardiovasc Diabetol.

[12] Pyrros A, Borstelmann SM, Mantravadi R, Zaiman Z, Thomas K, Price B, Greenstein E (2023). Opportunistic detection of type 2 diabetes using deep learning from frontal chest radiographs. Nat Commun.

[13] (2018). Social Deprivation Index (SDI). Robert Graham Center.

[14] Pope GC, Kautter J, Ellis RP, Ash AS, Ayanian JZ, Lezzoni LI, Ingber MJ (2004). Risk adjustment of medicare capitation payments using the CMS-HCC model. Health Care Financ Rev.

[15] Kaplan EL, Meier P (2012). Nonparametric estimation from incomplete observations. J Am Stat Assoc.

[16] Davidson-Pilon C (2019). Lifelines: survival analysis in Python. J Open Source Software.

[17] Uno H, Cai T, Pencina MJ, D'Agostino RB, Wei LJ (2011). On the C-statistics for evaluating overall adequacy of risk prediction procedures with censored survival data. Stat Med.

[18] Blanche P, Dartigues J, Jacqmin-Gadda H (2013). Estimating and comparing time-dependent areas under receiver operating characteristic curves for censored event times with competing risks. Stat Med.

[19] Blanche P, Proust-Lima C, Loubère L, Berr C, Dartigues J, Jacqmin-Gadda H (2015). Quantifying and comparing dynamic predictive accuracy of joint models for longitudinal marker and time-to-event in presence of censoring and competing risks. Biometrics.

[20] DeLong ER, DeLong DM, Clarke-Pearson DL (1988). Comparing the areas under two or more correlated receiver operating characteristic curves: a nonparametric approach. Biometrics.

[21] Schröder MS, Culhane AC, Quackenbush J, Haibe-Kains B (2011). Survcomp: an R/Bioconductor package for performance assessment and comparison of survival models. Bioinformatics.

[22] American Diabetes Association Professional Practice Committee for Diabetes* (2026). 2. Diagnosis and classification of diabetes: standards of care in diabetes-2026. Diabetes Care.

[23] Wilson PWF, Meigs JB, Sullivan L, Fox CS, Nathan DM, D'Agostino RB (2007). Prediction of incident diabetes mellitus in middle-aged adults: the Framingham offspring study. Arch Intern Med.

[24] Zhu Y, Dekker LH, Mierau JO (2023). Socio-economic gradients in diagnosed and undiagnosed type 2 diabetes and its related health complications. Nutr Metab Cardiovasc Dis.

[25] Hsueh L, Wu W, Hirsh AT, de Groot M, Mather KJ, Stewart JC (2020). Undiagnosed diabetes among immigrant and racial/ethnic minority adults in the United States: national health and nutrition examination survey 2011-2018. Ann Epidemiol.

[26] Cheng YJ, Kanaya AM, Araneta MRG, Saydah SH, Kahn HS, Gregg EW, Fujimoto WY (2019). Prevalence of diabetes by race and ethnicity in the United States, 2011-2016. JAMA.

[27] Hu MD, Lawrence KG, Bodkin MR, Kwok RK, Engel LS, Sandler DP (2021). Neighborhood deprivation, obesity, and diabetes in residents of the US gulf coast. Am J Epidemiol.

[28] (2022). National diabetes statistics report. Centers for Disease Control and Prevention.

[29] Khan SS, Matsushita K, Sang Y, Ballew SH, Grams ME, Surapaneni A, Blaha MJ (2024). Development and validation of the American heart association’s PREVENT equations. Circulation.

[30] Grundmann N, Mielck A, Siegel M, Maier W (2014). Area deprivation and the prevalence of type 2 diabetes and obesity: analysis at the municipality level in Germany. BMC Public Health.

[31] Christine PJ, Auchincloss AH, Bertoni AG, Carnethon MR, Sánchez BN, Moore K, Adar SD (2015). Longitudinal associations between neighborhood physical and social environments and incident type 2 diabetes mellitus: the multi-ethnic study of atherosclerosis (MESA). JAMA Intern Med.

[32] Xu J, Lawrence KG, O'Brien KM, Jackson CL, Sandler DP (2022). Association between neighbourhood deprivation and hypertension in a US-wide cohort. J Epidemiol Community Health.

[33] Deo SV, Al-Kindi S, Motairek I, Elgudin YE, Gorodeski E, Nasir K, Rajagopalan S (2023). Neighbourhood-level social deprivation and the risk of recurrent heart failure hospitalizations in type 2 diabetes. Diabetes Obes Metab.

[34] Kurani SS, Heien HC, Sangaralingham LR, Inselman JW, Shah ND, Golden SH, McCoy RG (2022). Association of area-level socioeconomic deprivation with hypoglycemic and hyperglycemic crises in US adults with diabetes. JAMA Netw Open.

