# Supplementary material for: Prediction of Type 2 Diabetes Mellitus From Chest X-Rays Using a Suite of Previously Developed Chronic Disease Deep Learning Models in an Ethnically Diverse Cohort: Observational Study
Source: JMIR AI. 2026 Jul 3;5:e85248. doi: 10.2196/85248 (PMC13379687; doi:10.2196/85248)
Supplement: Multimedia Appendix 1 [file ai_v5i1e85248_app1.docx]

APPENDIX 1

List of Medications Extracted as Indicative of T2D from 1/1/2010 to 9/12/2020

| albiglutide | glyburide | Linagliptin |
| --- | --- | --- |
| alogliptin | insulin aspart | lixisenatide |
| bexagliflozin | insulin aspart protamine | pioglitazone |
| canagliflozin | insulin degludec | repaglinide |
| chlorpropamide | insulin detemir | Ropinirole |
| Dapagliflozin* | insulin glargine | Rosiglitazone |
| Dulaglutide | insulin glulisine | Saxagliptin |
| Empagliflozin* | insulin isophane | Semaglutide |
| ertugliflozin | insulin lente | Sitagliptin |
| evolocumab | insulin lispro | Teduglutide |
| exenatide | insulin lispro protamine |  |
| glimepiride | insulin regular |  |
| glipizide | insulin ultralente |  |

*These meds were excluded after 4/30/2020 due to a new indication for CHF.

Metformin and liraglutide were excluded because they can be prescribed alone for indications unrelated to T2D, which could lead to misclassification.
