# Supplementary material for: Prediction of Type 2 Diabetes Mellitus From Chest X-Rays Using a Suite of Previously Developed Chronic Disease Deep Learning Models in an Ethnically Diverse Cohort: Observational Study
Source: JMIR AI. 2026 Jul 3;5:e85248. doi: 10.2196/85248 (PMC13379687; doi:10.2196/85248)
Supplement: Multimedia Appendix 2 [file ai_v5i1e85248_app2.docx]

APPENDIX 2

International Classification of Diseases for Diabetes Mellitus

| Classification | International Classification of Diseases, 9th Revision | International Classification of Diseases, 10th Revision |
| --- | --- | --- |
| Type I Diabetes Mellitus | 250.01, 250.03, 250.11, 250.13, 250.21, 250.23, 250.31, 250.33, 250.41, 250.43, 250.51, 250.53, 250.61, 250.63, 250.71, 250.73, 250.81, 250.83, 250.91, 250.93 | All E10.** |
| Type II Diabetes Mellitus | 250.00, 250.02, 250.10, 250.12, 250.20, 250.22, 250.30, 250.32, 250.40, 250.42, 250.50, 250.52, 250.60, 250.62, 250.70, 250.72, 250.80, 250.82, 250.90, 250.92 | All E11.** |
