# Supplementary material for: Prediction of Type 2 Diabetes Mellitus From Chest X-Rays Using a Suite of Previously Developed Chronic Disease Deep Learning Models in an Ethnically Diverse Cohort: Observational Study
Source: JMIR AI. 2026 Jul 3;5:e85248. doi: 10.2196/85248 (PMC13379687; doi:10.2196/85248)
Supplement: Multimedia Appendix 3 [file ai_v5i1e85248_app3.docx]

APPENDIX 3

Euler diagram showing the percentage of the cohort identified with Type 2 Diabetes based on medications (Appendix 1), HbA1c ≥ 6.5%, and ICD-9/ICD-10 codes (Appendix 2). Overlapping regions indicate patients meeting multiple criteria.


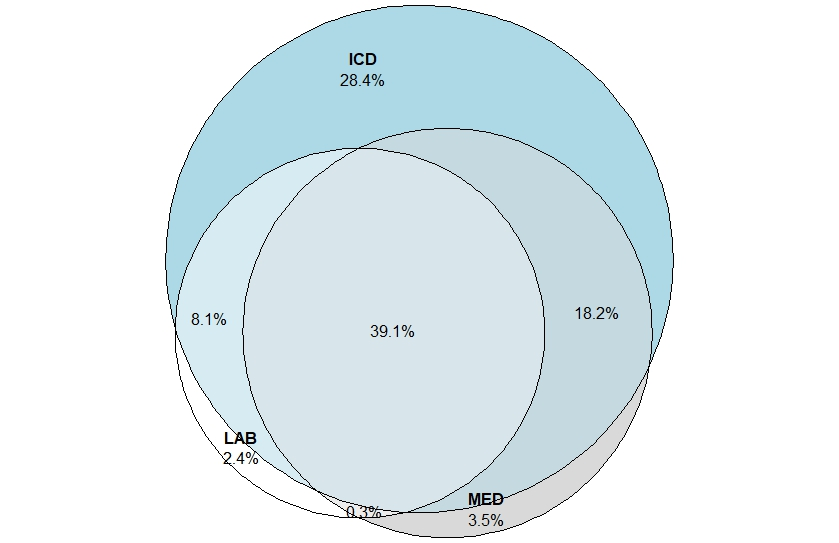


Data from the whole cohort with all positive results regardless of the timing of the CXR. The addition of HbA1c results to ICD codes added 2.8% patients. The use of a limited set of diabetic medications (Appendix 1) added 4.1% patients. The use of both HbA1c and meds together added 6.6% patients. The calculated AUC in these cohorts was not different to the 3^rd^ digit, AUC 0.805.
