# Supplementary material for: Prediction of Type 2 Diabetes Mellitus From Chest X-Rays Using a Suite of Previously Developed Chronic Disease Deep Learning Models in an Ethnically Diverse Cohort: Observational Study
Source: JMIR AI. 2026 Jul 3;5:e85248. doi: 10.2196/85248 (PMC13379687; doi:10.2196/85248)
Supplement: Multimedia Appendix 4 [file ai_v5i1e85248_app4.docx]

APPENDIX 4

Calibration Curves(a) and Measures(b) for Prevalence by Age, BMI and Race

a)


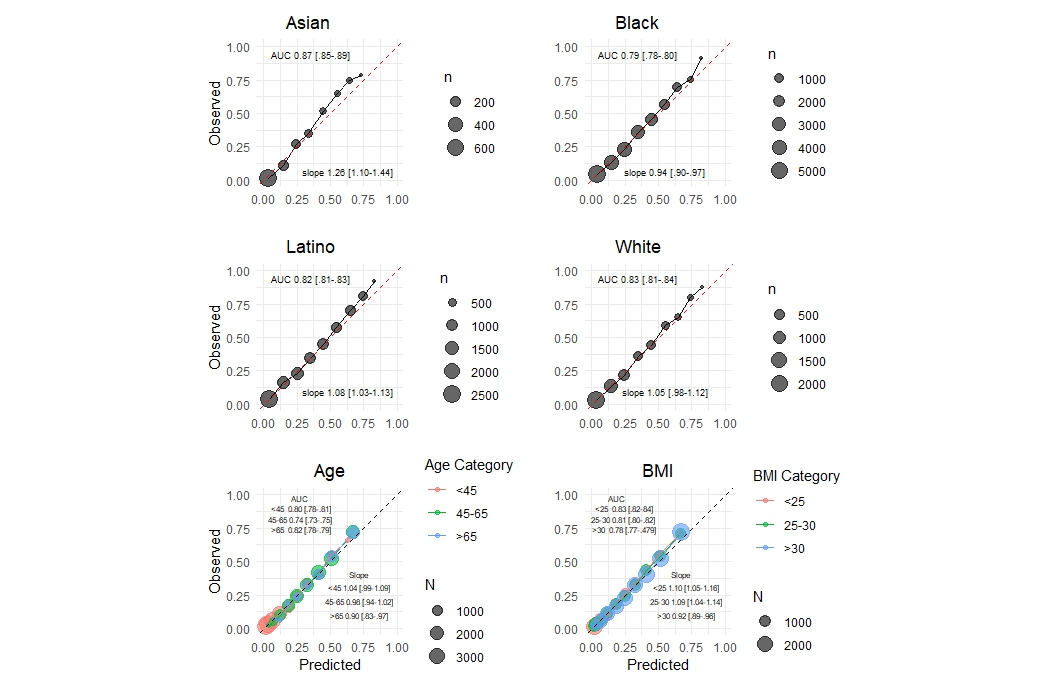


b)

|  | N | Calibration_slope | Calibration_intercept | Brier_score | ECE^**^ | AUC |  |
| --- | --- | --- | --- | --- | --- | --- | --- |
| Race |  |  |  |  |  |  |  |
| Black | 21306 | 0.94 [.90-.97] | -0.018 | 0.155 | 0.022 | 0.79 [.78-.80]^*^ |  |
| White | 5597 | 1.05 [.98-1.12] | 0.093 | 0.127 | 0.015 | 0.83 [.81-.84]^*^ |  |
| Latino | 9161 | 1.08 [1.03-1.13] | 0.088 | 0.158 | 0.018 | 0.82 [.81-.83]^*^ |  |
| Asian | 1320 | 1.26 [1.10-1.44] | 0.343 | 0.103 | 0.029 | 0.87 [.85-.89]^*^ |  |
| Age |  |  |  |  |  |  |  |
| <45 | 18496 | 1.04 [.99-1.09] | -0.010 | 0.084 | 0.009 | 0.80 [.78-.81] |  |
| 45-65 | 21367 | 0.98 [.94-1.02] | -0.041 | 0.186 | 0.019 | 0.74 [.73-.75] |  |
| >65 | 386 | .90 [.83-.97] | -0.055 | 0.210 | 0.021 | 0.82 [.76-.88] |  |
| BMI |  |  |  |  |  |  |  |
| <25 | 10632 | 1.10 [1.05-1.16] | -0.007 | 0.038 | 0.009 | 0.83 [.82-.84] |  |
| 25-30 | 11260 | 1.09 [1.04-1.14] | -0.002 | 0.058 | 0.009 | 0.81 [.80-.82] |  |
| >30 | 18438 | 0.92 [.89-.96] | -0.026 | 0.166 | 0.015 | 0.78 [.77-.79] |  |

*All AUCs compared using Delong method and Bonferroni-Holm correction with P < 0.003, except Latino vs. White
 which were not different, P=0.5
** ECE is Expected Calibration Error
