# Supplementary figures and images for: Prediction of Type 2 Diabetes Mellitus From Chest X-Rays Using a Suite of Previously Developed Chronic Disease Deep Learning Models in an Ethnically Diverse Cohort: Observational Study
Source: JMIR AI. 2026 Jul 3;5:e85248. doi: 10.2196/85248 (PMC13379687; doi:10.2196/85248)

APPENDIX 5


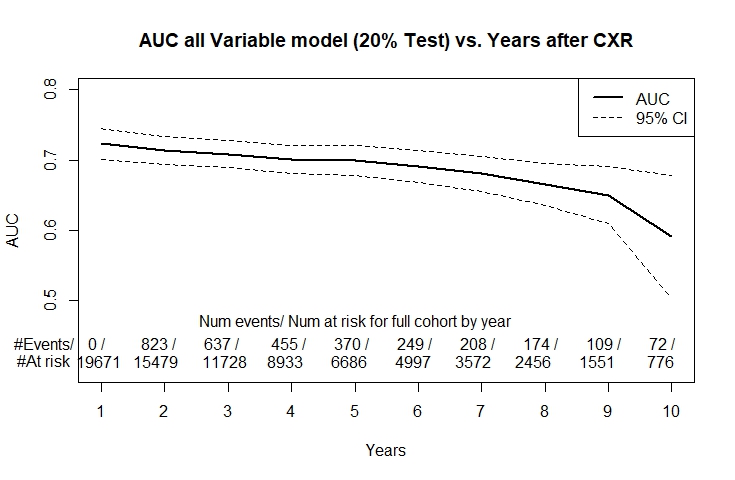

Supplement: Multimedia Appendix 5 [file ai_v5i1e85248_app5.docx]
