# Supplementary material for: Prediction of Type 2 Diabetes Mellitus From Chest X-Rays Using a Suite of Previously Developed Chronic Disease Deep Learning Models in an Ethnically Diverse Cohort: Observational Study
Source: JMIR AI. 2026 Jul 3;5:e85248. doi: 10.2196/85248 (PMC13379687; doi:10.2196/85248)

Appendix 6

Distribution of SDI in Prevalence Cohort

Shown below is the number of patients for each SDI score from 1 to 100 in the prevalence data set, N= 39,908. The first quartile ends at 68, noted as Q1. The fourth quartile begins at 97, noted as Q4. The Median is 92, noted with a vertical red line. The interquartile range (IQR) is from 68-97 or 29. This is noted with beige shading.


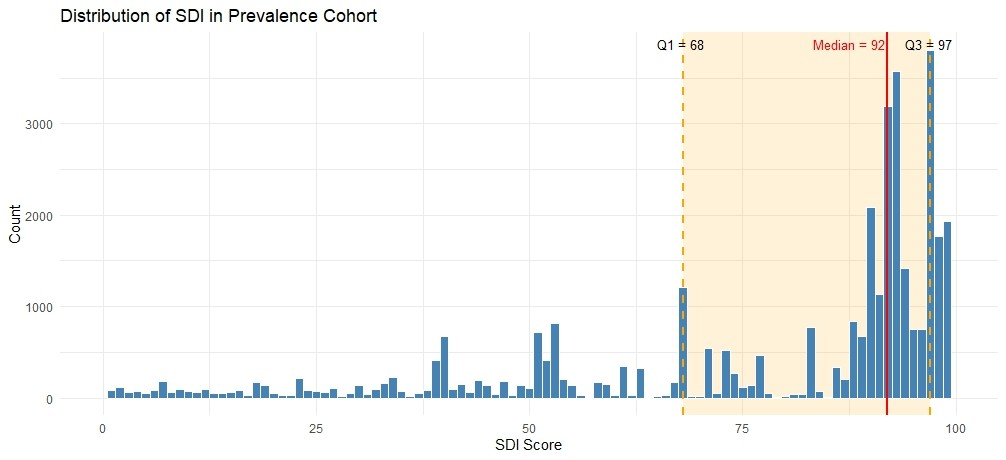

Supplement: Multimedia Appendix 6 [file ai_v5i1e85248_app6.docx]
